# Seroprevalence of SARS-CoV-2 IgG Antibodies in Corsica (France), April and June 2020

**DOI:** 10.3390/jcm9113569

**Published:** 2020-11-05

**Authors:** Lisandru Capai, Nazli Ayhan, Shirley Masse, Jean Canarelli, Stéphane Priet, Marie-Hélène Simeoni, Remi Charrel, Xavier de Lamballerie, Alessandra Falchi

**Affiliations:** 1 Laboratoire de Virologie, UR 7310, Université de Corse, 20250 Corte, France; nazliayhann@gmail.com (N.A.); masse_s@univ-corse.fr (S.M.); falchi_a@univ-corse.fr (A.F.); 2 Unité des Virus Émergents (UVE), Aix Marseille Univ, IRD 190, INSERM 1207, IHU Méditerranée Infection, 13005 Marseille, France; stephpriet@gmail.com (S.P.); remi.charrel@univ-amu.fr (R.C.); xavier.de-lamballerie@univ-amu.fr (X.d.L.); 3 Laboratoire de Biologie Médicale CCF, associated with UR7310, Université de Corse, 20000 Ajaccio, France; jeancanarelli@gmail.com; 4 Laboratoire de Biologie Médicale 2A2B, associated with UR7310, Université de Corse, 20250 Corte, France; marie-helene.simeoni@orange.fr

**Keywords:** seroprevalence, SARS-CoV-2, antibodies, ELISA, seroneutralization, France

## Abstract

Our aim was to assess the seroprevalence of severe acute respiratory syndrome coronavirus-2 (SARS-CoV-2) infection after the lockdown in a sample of the Corsican population. Between 16 April and 15 June 2020, 2312 residual sera were collected from patients with a blood analysis conducted in one of the participating laboratories. Residual sera obtained from persons of all ages were tested for the presence of anti-SARS-CoV-2 Immunoglobulin G (IgG) using the EUROIMMUN enzyme immunoassay kit for semiquantitative detection of IgG antibodies against the S1 domain of viral spike protein (ELISA-S). Borderline and positive samples in ELISA-S were also tested with an in-house virus neutralization test (VNT). Prevalence values were adjusted for sex and age. A total of 1973 residual sera samples were included in the study. The overall seroprevalence based on ELISA-S was 5.27% (95% confidence interval (CI), 4.33–6.35) and 5.46% (4.51–6.57) after adjustment. Sex was not associated with IgG detection. However, significant differences were observed between age groups (*p*-value = 1 E-5). The highest values were observed among 10–19, 30–39, and 40–49 year-old age groups, ranging around 8–10%. The prevalence of neutralizing antibody titers ≥40 was 3% (2.28–3.84). In conclusion, the present study showed a low seroprevalence for COVID-19 in Corsica, a finding that is in accordance with values reported for other French regions in which the impact of the pandemic was low.

## 1. Introduction

On 30 December 2019, the Municipal Health Commission in Wuhan (Hubei province, China) reported a cluster of unexplained pneumonia cases [1]. In January 2020, a betacoronavirus named the severe acute respiratory syndrome coronavirus (SARS-CoV-2) was identified [2,3]. The disease caused by the SARS-CoV-2 was named coronavirus infectious disease 2019 (COVID-19). COVID-19 is a highly infectious disease and, following the first cases in China, the virus spread rapidly worldwide. Reasons for the rapid spread of SARS-CoV-2 include the high transmissibility of the virus [4], asymptomatic or paucisymptomatic carriers [5], and the lack of any apparent cross-protective immunity from related viral infections [6]. On 30 January 2020, the World Health Organization (WHO) declared a Public Health Emergency of International Concern [7]. As of 4 September 2020, the number of SARS-CoV-2 confirmed cases exceeded 26million with more than 800,000 reported deaths. The socioeconomic impact of the COVID-19 pandemic has also been significant, with lockdowns drastically reducing the mobility and productivity of much of the world’s population [8].

In France, the SARS-CoV-2 epidemic period ranged from 2020w09 (24–29 February) to 2020w19 (4–10 May), with an epidemic peak at 2020w13 (23–29 March) and a positivity rate of up to 30%, affecting mostly the Paris and northeastern regions of the country [9]. To respond to the epidemic, on 17 March (2020w12), France ordered all non-essential retailers and services to be closed, and the general population to remain confined. These measures have reduced the number of incident cases and the stress on the health care system. The French government announced the end of the lockdown on 11 May (2020w21). After the epidemic period, it has been projected that in France, 3.7 million (range: 2.3–6.7) people, i.e., 5.7% of the population, will have been infected [10].

Corsica, a French Mediterranean island, was affected by the COVID-19 pandemic, like continental France, at the beginning of March. The SARS-CoV-2 positivity rate ranged from 3.5% (2020w09) to 2.0% (2020w19), and peaked during 2020w13 at 14% [11]. As of 12 May, 55 people had died (46 in South Corsica, 9 in Haute-Corse) of COVID-19 in hospital (mortality rate = 0.0002% and lethality rate = 21.6%).

These epidemiological data included only a fraction of the real number of SARS-CoV-2 infections, since not all infected patients were symptomatic, required hospitalizations, or provided specimens for laboratory testing [12]. The capacity to estimate the spread of SARS-CoV-2 depends on our knowledge of the immune status against SARS-CoV-2 in the population.

The primary outcome of this study was to estimate for the first time the prevalence of Immunoglobulin G (IgG) antibodies against SARS-CoV-2 in the Corsican population to improve epidemiological knowledge of the virus spread and to estimate what part of the Corsican population has been infected by the SARS-CoV-2. The secondary outcome was to estimate neutralizing antibodies against SARS-CoV-2 by using an in-house virus neutralization test (VNT) currently considered to be the most specific and sensitive serological assay capable of evaluating and detecting functional neutralizing antibodies. Monitoring of seroprevalence can guide public health measures to fight the pandemic.

## 2. Experimental Section

### 2.1. Study Area and Population

**Figure A1:**
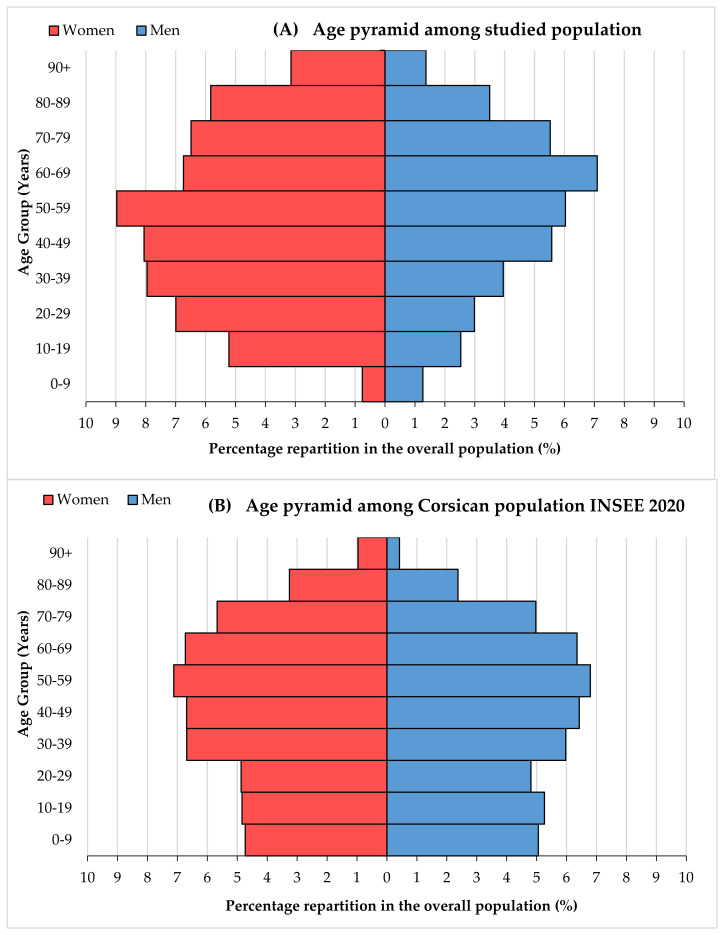
Age and sex repartition pyramid among Corsican population (**A**) and studied population (**B**).

The study was conducted on the French Mediterranean island of Corsica (8680 km^2^) located south-east of mainland France. The population of the island was estimated at 344,679 as of 1 January 2019 [13]. This region is composed of two administrative departments (Haute-Corse and Corse-du-Sud) and five districts (Ajaccio, Bastia, Calvi, Corte and Sartène) including 365 counties. The age and sex distribution by age groups of the Corsican population was obtained from the French National Institute of Statistics and Economic Studies (INSEE) [13] (Appendix A; Figure A1).

### 2.2. Sample Size

The sample size was calculated according to epidemiological calculation tools [14]. A minimum sample size of 1814 was calculated assuming an a priori 5% IgG anti-SARS-CoV-2 seroprevalence [10], a confidence in the estimate of 95%, a maximum allowable error in the prevalence of 1%, and a Corsican population size of 344,679 habitants based on the latest French census data [13].

### 2.3. Studied Population

The sampling plan was established considering the minimum estimated sample size and the actual distribution of the Corsican population by age group and sex. Information about the distribution of the studied population and the general population of Corsica by age group and sex is available in the Appendix (Figure A1A,B).

**Figure 1:**
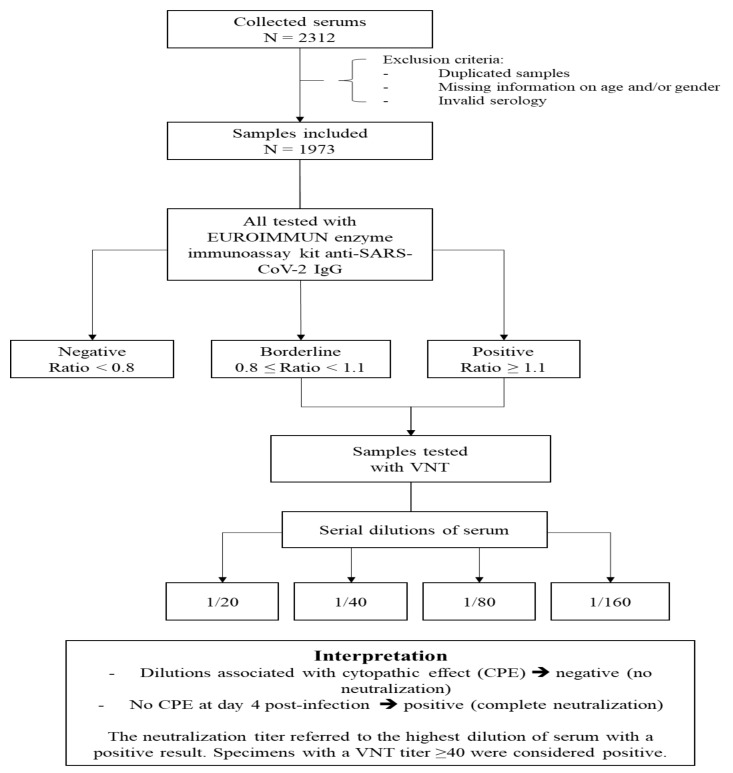
Diagram on sample inclusion and analyses performed. VNT: Virus neutralization test

Between 16 April and 15 June 2020, 2312 residual sera were collected from patients with a blood analysis conducted in one of the two participating laboratories (Figure 1). The conditions of exclusion were the following: one or more samples from the same person (only one sample was included in the study) and/or sample with missing information on age and/or sex and/or invalid serology result (insufficient serum volume or invalid result). The two private participating laboratories are located in Ajaccio and Corte. Ajaccio, located in the south-west, is one of the two largest cities in Corsica and corresponds to the area of the island where the vast majority of COVID-19 cases were detected during the SARS-CoV-2 epidemic. The laboratory located in Ajaccio is one of the island’s main laboratories and analyzes samples coming from the southern and north-western regions of the island. The Laboratory of Corte analyzes samples from the center of Corsica.

### 2.4. Serological Analyses

Residual sera obtained from persons of all ages were tested for the presence of anti-SARS-CoV-2 IgG using the EUROIMMUN enzyme immunoassay kit for semiquantitative detection of IgG antibodies against the S1 domain of a viral spike protein (ELISA-S) (reference: EI 2606–9601 G; EUROIMMUN, Bussy-Saint Martin, France). Wells were coated with recombinant structural protein of SARS-CoV-2. This immunoassay was chosen because it has already been evaluated by numerous studies [15,16,17,18,19,20] and is on the list of authorized serological tests for the national health authorities in France.

For each sample, the optical density (OD) ratio was estimated. According to the manufacturer’s instruction, a result was considered borderline if the ratio was between ≥0.8 and <1.1 and positive if the sample ratio was ≥1.1. Positive and negative controls were performed on each ELISA plate and were valid.

In all the samples with a ratio ≥0.8, neutralizing antibodies were detected using a VNT as previously described [21]. VeroE6 cells cultured in 96-well microplates, 100 Fifty-percent tissue culture infective dose (TCID_50_) of the SARS-CoV-2 strain BavPat1 (courtesy of Pr. Drosten, Berlin), and serial dilutions of serum (1/20–1/160) were used. Dilutions associated with cytopathic effect (CPE) were considered negative (no neutralization) and those with no CPE at day 4 post-infection were considered positive (complete neutralization). The neutralization titer refers to the highest dilution of serum with a positive result. Specimens with a VNT titer ≥40 were considered positive [21] (Figure 1).

### 2.5. Ethical Statement

No nominative or sensitive data on participants were collected. This seroprevalence study f within the scope of the French Reference Methodology MR-004 according to Law 2016-41 dated 26 January 2016 on the modernization of the French health system.

### 2.6. Collection Data and Statistical Analysis

According to French Reference Methodology MR-004, the regulation on research projects concerning tube bottoms, only the age, sex, date, and laboratory place of collection could be collected for each sample. The statistical analyses were performed for these variables.

Descriptive statistical methods were performed for age and sex. Age is described as median with interquartile ranges (IQRs). Age groups were categorized as follows: 0–9, 10–19, 20–29, 30–39, 40–49, 50–59, 60–69, 70–79, 80–89, and ≥90 years. All categorical data are reported as percentages. IgG anti-SARS-CoV-2 seroprevalences and its 95% exact binomial confidence intervals (CIs) were estimated. Associations of the presence of anti-SARS-CoV-2 IgG with sex and age and location were analyzed and tested using the χ2 test or Fisher’s exact test. Statistical significance was set at a *p*-value < 0.05. The odds ratio (OR) was used to describe the risk of different age groups and sex in positive ELISA-S serums compared with non-positive ELISA-S serums. Seroprevalence by age group and sex was adjusted according to the proportions observed in the real population [13]. This adjustment was performed by specifically weighting each individual. The following R packages were used for the statistical analysis: questionr, car, stats, survey, FactoMineR, and ade4. All statistical analyses were performed using R software version 3.6.1 (R Foundation, Vienna, Austria).

## 3. Results

A total of 1973 sera of patients were included from the two participating private medical laboratories between late April and June 2020. People included ranged from 6 months to 101 years of age, including 1187 women (60.2%) and 786 men (39.8%). The median and the mean age were 52 years (interquartile range (IQR) = 34–70). The weighted population according to sex and age groups showed that the mean age was 44 years, including 51.7% women and 48.3% men.

### 3.1. Seroprevalence Estimated with Results of the EUROIMMUN ELISA IgG Anti-SARS-CoV-2

**Figure 2:**
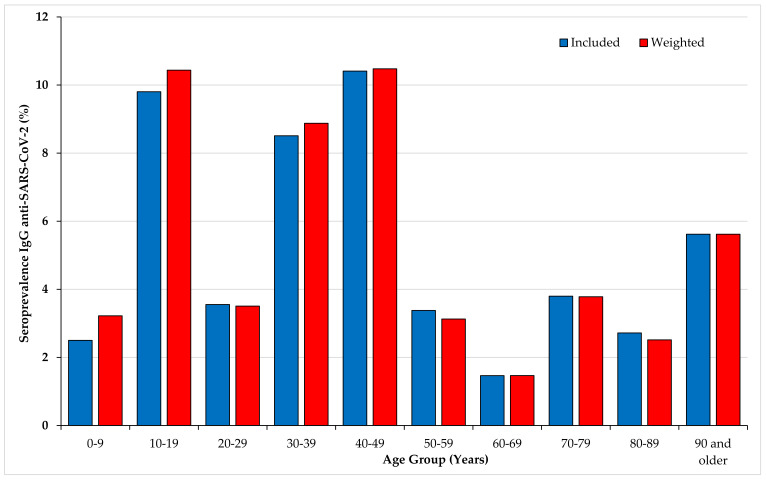
Seroprevalences of IgG anti-SARS-CoV-2 by age groups (included and weighted population). IgG: Immunoglobulin G; SARS-CoV-2: severe acute respiratory syndrome coronavirus-2.

Table 1 and Figure 2 describe the overall seroprevalence and the seroprevalence rates estimated by age groups and sex in the included and in the weighted population. 104 of 1973 collected samples were found positive for anti-SARS-CoV-2 (index ≥1.1). The overall seroprevalence in our study population was 5.27% [4.33–6.35] and 5.46% [4.51–6.57] after adjustment. The median age among the positives was 41 years and the mean age was 44.92 years. Gender was not associated with the seroprevalence rate. In contrast, univariate analysis showed significant seroprevalence rate differences by age groups (*p*-value = 1 E-5). The highest rate was observed in adults aged 40–49 (10.48%), followed by 10–19 (10.44%) and 30–39 (8.88%). The seroprevalence values among the 0–9 year-olds and 20–29-year-olds were significantly lower with respect to the seroprevalence reported in the 10–19, 30–39, and 40–49 age groups (*p* = 0.004 and 0.03, respectively). Individuals younger than 50 years of age had a seroprevalence rate significantly higher (7.60%) than people older than 50 (2.80%) (OR = 2.86; 95% confidence intervals (CIs):1.80–4.53; *p* < 0.000001.)

### 3.2. Results of VNT Assay

Among the 140 samples with an ELISA ratio ≥0.80 (Table 2), 104 samples showed a ratio >1.1 and 36 showed a ratio ranging from 0.8 to 1.1. Forty-two percent (*n* = 59) of 140 samples had a positive neutralization antibody titer (VNT titer ≥40) (Table 2).

Among the 104 ELISA with a ratio >1.1, 53.8% (*n* = 56) had a VNT titer ≥40. Among the 36 samples with an ELISA ratio between 0.80 and 1.1, 91.6% (*n* = 33) had a VNT titer <40 and 8.3% (*n* = 3) had a positive VNT titer ≥40. VNT titers did not differ significantly among age groups and similar VNT values were observed between men and women. The overall prevalence of samples above the cut-off (titer 40) (59/1973) was 3% [2.28–3.84].

## 4. Discussion

To the best of our knowledge, this is the first study describing the prevalence of SARS-CoV-2 antibodies in a representative sample of Corsican patients with a blood analysis performed in biological laboratories after the COVID-19 epidemic period.

The seroprevalence value estimated in the present study with ELISA-S (5.46% [4.51–6.57]; approximately 18,800 people) is in line with an estimation that 3.7 million (range: 2.3–6.7) people, i.e., 5.7% of the French population, will be infected during the epidemic period [10]. The rate of seroprevalence reported in Corsica is closer to the rate reported by a similar study in a French region with a low proportion of COVID-19 cases during the epidemic period (3%) than those reported in two regions with the highest rates (9–10%) [22]. The observed seroprevalence in our population is in line with ELISA-S values reported in Spain, New York City, and in different subcohorts of Wuhan [23], the United States [24], and China [25], but lower than values reported in heavily affected areas such as Switzerland [26], northern Italy [27], and the urban areas around Madrid [28].

In the present study, we did not observe a significant distribution of seroprevalence values between men and women, in agreement with previous reports in Spain [23], Dutch blood donors [29], and French blood donors [21]. This is in line with sex-disaggregated data for COVID-19 in several European countries showing a similar number of cases between the sexes but more severe outcomes in aged men [30]. We observed that seroprevalence values differed significantly among age groups. High seroprevalence values, ranging around 8–10%, were observed among 10–19, 30–39, and 40–49 year-old age groups. This is in line with results previously reported by a population-based serosurvey in Geneva [26] and with the values reported in three French general populations [22]. In our sample, the seroprevalence values suggest that infection was less prevalent in children than in adolescents during the epidemic period. The lower prevalence in children might be related in part to lower nasal gene expression of angiotensin-converting enzyme 2 [31].

Because the ELISA assay could exhibit cross-reactivity with antibodies to other seasonal human coronaviruses, some results may represent false positives, leading to overestimation of seroprevalence data [32]. The EUROIMMUN assay used in this study was evaluated in different studies showing a specificity ranging from 96.2% to 100% and sensitivity ranging from 86.4% to 100% [15,17,33]. The sensitivity and specificity of the ELISA can vary considerably depending on the timing of the sample, in particular from samples collected ≥4 days after COVID-19 diagnosis by PCR. The sensitivity can increase from 67% to 100% between samples analyzed at the time of PCR and samples analyzed at least four days later [15]. VNT, currently considered the gold standard, is the most specific serological assay capable of detecting true positive cases and functional neutralizing antibodies [34]. The positivity of a titer ≥40 for the VNT assay used in the present study is an indicator of strong specificity (100%) [21]. Around half of the samples showing ELISA results ≥0.8 and half showed a VNT ≥40, in line with a previous study [22]. The prevalence value based on our VNT analysis revealed that at least 3% of the included samples have been exposed to the virus and had developed a titer of neutralizing antibodies ≥40. This value is in line with the low value of 2.7% observed with the same VNT assay in blood donors collected during the last week of March or the first week of April 2020 in four French departments [21] and in the general adult population of a region with low prevalence [22].

Seroprevalence data based on the ELISA assay provided information about previous exposure to SARS-CoV-2 but not about protection. In the present study, we also estimated SARS-CoV-2 neutralizing antibody titers among serum samples with dubious (≥0.8 and <1.1) and positive (≥1.1) results. About half of these samples had neutralizing antibodies with a titer ≥40. This represents a preliminary approach to protection against re-infection but no precise correlate of protection is available yet [35].

Another interesting point to discuss at the epidemiological level is the waning antibody level in asymptomatic and symptomatic SARS-CoV-2 infections. Different studies have reported this decrease in the months following infection [36,37,38]. Most convalescent people who recover from COVID-19 do not have high levels of neutralizing activity. This possible loss of immunity over time must therefore be considered when interpreting seroprevalence studies for SARS-CoV-2. Moreover, if the objective is to estimate the number of infections, it is preferable to conduct seroprevalence studies as close as possible in time to the epidemic of interest when most infected individuals will still have easily detectable SARS-CoV-2 antibodies to better estimate the true number of infections.

There are several limitations to our study. Firstly, residual sera from screening or routine care provided by private medical biology laboratories are more likely to come from people needing to monitor their health status for chronic medical conditions. Thus, these data cannot be extrapolated to the general population, although we adjusted the data according to the age and sex of the general Corsican population. Additionally, no data concerning clinical features, chronic disease, or possible COVID-19 exposures were available, thereby potentially biasing results. This lack of information on the COVID-19 status of the persons included could also have influenced the specificity and sensitivity of the ELISA test (timing of the sample in relation to the infection). As we tested only seroneutralization samples with an ELISA-S test optical density of ≥0.8, seroprevalence could be slightly underestimated. The strengths of this study were the size of the sample and its representativeness in terms of age and gender. Samples were analyzed by combining ELISA and neutralization methods to strengthen results.

In conclusion, the present study showed that the low seroprevalence for COVID-19 in Corsica is in accordance with values reported for other French regions in which the impact of the pandemic was low. This regional study is particularly important in Corsica as the island situation cannot be extrapolated from neighboring regions.

## Figures and Tables

**Table 1:** Description of the population (included and adjusted) and univariate analysis of association with IgG anti-SARS-CoV-2 seropositivity (*p*-value and odds ratio (OR)).

Items		Included Population						Weighted Population					
		All (*n*)	EUROIMMUN IgG SARS-CoV-2 Positives			OR [95% CI]	*p*-Value	All (*n*)	Weighted Seroprevalences			OR [95%CI]	*p*-Value
			*n* Positive	SARS-CoV-2 IgG+ (%)	[95% CI]				*n* Positives	Seroprevalence (%)	[95% CI]		
Overall		1973	104	5.27	[4.33–6.35]			1973	107.66	5.46	[4.37–7.00]		
Sex	Women	1187	66	5.56	[4.33–7.02]	0.86 [0.57–1.29]	0.48	1017.35	58.41	5.74	[4.31–7.17]	0.89 [0.56–1.42]	0.63
	Men	786	38	4.83	[3.44–6.58]			955.48	49.25	5.15	[3.75–6.56]		
Age (years)	0–9	40	1	2.50	[0.063–13.16]	0.73 [0.04–3.99]	< 0.0001 *	193.05	6.22	3.22	[0.73–5.71]	1.03 [0.13–8.36]	0.97
	10–19	153	15	9.80	[5.59–15.65]	3.11 [1.38–7.32] *		199.79	20.85	10.44	[6.20–14.68]	3.62 [1.54–8.53] *	0.0033 *
	20–29	197	7	3.55	[1.44–7.18]	1.05 [0.38–2.79]		191.59	6.72	3.51	[0.90–6.10]	1.13 [0.40–3.19]	0.82
	30–39	235	20	8.51	[5.27–12.84]	2.66 [1.25–6.04] *		249.66	22.16	8.88	[5.35–12.40]	3.01 [1.35–6.69] *	0.0069 *
	40–49	269	28	10.41	[7.03–14.69]	3.32 [1.63–7.32] *		258.47	27.08	10.48	[6.74–14.21]	3.62 [1.71–7.68] *	0.00079 *
	50–59	296	10	3.38	[1.63–6.12]	reference		274.30	8.58	3.13	[1.07–5.19]	reference	
	60–69	273	4	1.47	[0.40–3.71]	0.43 [0.12–1.29]		257.60	3.78	1.47	[0.00–2.94]	0.46 [0.14–1.50]	0.20
	70–79	237	9	3.80	[1.75–7.09]	1.13 [0.44–2.85]		209.46	7.92	3.78	[1.20–6.36]	1.21 [0.48–3.05]	0.68
	80–89	184	5	2.72	[0.89–6.23]	0.80 [0.25–2.29]		111.32	2.80	2.52	[0.00–5.42]	0.79 [0.26–2.36]	0.68
	90 and older	89	5	5.62	[1.85–12.62]	1.70 [0.52–4.93]		27.59	1.55	5.62	[0.00–14.21]	1.80 [0.59–5.49]	0.30

SARS-CoV-2: severe acute respiratory syndrome coronavirus-2; IgG: Immunoglobulin G; OR: Odd Ratio; CI: Confidential Interval; * the asterisk means that the figures concerned are significant (*p*-value < 0.05) and OR not including 1.

**Table 2:** Virus neutralization test (VNT) results of 140 samples with an ELISA ratio >0.8.

VNT Titer	Number of Samples	%	VNT Interpretation *n* (%)
Negative at titer 20	67	47.9	Beside the cut-off (titer 40) 81 (58.0%)
20	14	10.0	
40	24	17.1	59 (42.0%)
80	9	6.4	
160	26	18.6	
Total	140	100
